# Pyroptosis Patterns Are Involved in Immune Microenvironment Regulation of Dilated Cardiomyopathy

**DOI:** 10.1155/2022/4627845

**Published:** 2022-03-10

**Authors:** Kexin Wang, Zhan Lv, Chenggang Fang, Chengkai Xu, Zhimin Yu, Liuming Gao, Yanggan Wang

**Affiliations:** ^1^Department of Internal Medicine, Zhongnan Hospital of Wuhan University, Wuhan, China; ^2^Medical Research Institute of Wuhan University, Wuhan, China

## Abstract

The importance of cell pyroptosis in immunity regulation is becoming increasingly obvious, especially in diseases of the cardiovascular system. Nevertheless, it is unknown whether the pyroptosis signalling pathway is involved in the immune microenvironment regulation of dilated cardiomyopathy (DCM). Therefore, the purpose of the study was to investigate the influence of pyroptosis on the immune environment in dilated cardiomyopathy. We found that expression of 19 pyrolysis-related genes (PRGs) in DCM samples was altered compared to healthy samples. Subsequently, based on these 12 hub pyrolysis-related genes, we developed a classifier that can distinguish between healthy samples and DCM samples. Among the hub pyrolysis-related genes, RT–PCR analyses demonstrated that five of them exhibited significant differential expression in DCM. Interestingly, we observed that immune characteristics are correlated with pyroptosis: higher expression of GSDMD is positively correlated with infiltrating activated pDCs; GSDMD is negatively correlated with Tregs; CASP1 is positively related to parainflammation; and CASP9 is negatively related to the type II IFN response. In addition, distinct pyroptosis-mediated patterns were identified, and immune characteristics under distinct patterns were revealed: pattern B mediates an active immune response, and pattern A leads to a relatively mild immune response to DCM. We also compared the biological functions between these patterns. Compared with pattern A, pattern B had more abundant pathways, such as the NOTCH signalling pathway and pentose phosphate pathway. In summary, this study proves the important influence of pyrolysis on the immune microenvironment of dilated cardiomyopathy and provides new clues for understanding the pathogenesis of dilated cardiomyopathy.

## 1. Introduction

Dilated cardiomyopathy (DCM) is a very common myocardial disease, and it is estimated that 1 in 250 people is affected [1, 2]. Dilated cardiomyopathy (DCM) is characterized by an enlargement in either the left or both ventricles, accompanied by myocardial hypertrophy and decreased ventricular systolic function that may also include congestive heart failure [3]. DCM progressively worsens and can easily develop into heart failure. Death can occur at any stage of DCM and is the most common indication for heart transplantation. DCM portends a poor prognosis and is one of the major indications for cardiac transplantation [4]. The causes of DCM are heterogeneous and can result from idiopathic, genetic, viral, immune, or toxic aetiology [1]. DCM is caused by a combination of genetic and environmental factors in the myocardium [1, 5]. During the progression of DCM, immune function often becomes disordered, affecting both cellular immunity and humoral immunity. Inflammatory endothelial activation is often present in DCM, with lymphocyte and monocyte infiltration [6–8]. Consequently, it is important to explore the immune molecular pathways of DCM to understand the pathological mechanisms underlying it; at the same time, these immune targets may inspire ideas for the design of therapeutic strategies in DCM.

Classically, there are three mechanisms of cell death: apoptosis, autophagic cell death, and necrosis. Recent studies have found that caspase-1 in both humans and mice, caspase-4/5 in humans, and caspase-11 in mice mediate a new type of programmed necrosis, pyroptosis [9, 10]. Under the electron microscope, before rupture of the cell plasma membrane, pyrolyzed cells can be seen forming a large number of vesicles, namely, inflammasomes. Then, pores are formed in the cell membrane, which causes subsequent rupture and release of the contents [11]. These are the morphological features of cellular pyroptosis. Pyroptosis is a form of gasdermin-mediated programmed cell necrosis [12, 13]. Pyrolysis is an important natural immune response in the body that plays a significant role in the fight against infection [12]. Recently, extensive findings have indicated that pyroptosis is involved in various diseases, especially cardiovascular diseases (CVDs) [14, 15]. For instance, in atherosclerosis, ischaemia–reperfusion injury, myocardial infarction, coronary calcification, and heart failure, related research results have led to the discovery and application of inhibitors or drugs targeting proteins involved in pyroptosis [16].

Nevertheless, few studies have focused on the mechanisms and pathways related to cell pyroptosis in dilated cardiomyopathy (DCM). Zeng et al. [17] demonstrated that in the mechanism of DCM, the NLRP3 inflammasome plays a critical role by activating caspase-1 and leading to pyroptosis. However, it is unknown whether the pyroptosis pathway is mechanistically related to the immune microenvironment in dilated cardiomyopathy.

In this study, pyroptosis patterns in DCM were systematically investigated. We found that pyroptosis-related genes could differentiate DCM samples from healthy samples. We found that the abundance of infiltrating immune cells and the immune response gene set in DCM exhibited linear pertinence, showing a strong relationship between pyroptosis-related genes and immune regulation. We analysed DCM samples based on 12 pyroptosis-related genes, and 2 distinct pyroptosis patterns were identified. Between these subtypes, we observed different characteristics in immune regulation, and biological functions were compared between these subtypes. In addition, we analysed 2142 pyroptosis-related genes and their biological functions. Our results suggest that cellular pyroptosis patterns make a critical contribution to the immune microenvironment in DCM.

## 2. Materials and Methods

### 2.1. Data Preprocessing

We downloaded the GSE141910 dataset (http://ncbi.nlm.nih.gov/geo/query/acc.cgi?acc=GSE141910) from the Gene Expression Omnibus (GEO) database, which included 166 healthy samples and 116 DCM samples. All data were preprocessed and obtained using the R package “GEOquery.” The GPL16791 platform file was used for annotation. Gene probes were annotated with gene symbols, and probes that did not match gene symbols or that matched multiple symbols were excluded. We collected 33 PRGs from previous research [18–21].

### 2.2. Analysis of Changes in PRGs between DCM and Healthy Samples

To explore interactions among the 29 PRGs, we used the Search Tool for the Retrieval of Interacting Genes/Proteins (STRING) database (http://www.string-db.org/) to create a protein–protein interaction (PPI) network of these PRGs. We used Spearman correlation analysis to evaluate the expression relationships among 29 PRGs in all samples and specifically in DCM samples, and using the Wilcoxon test, we compared the expression differences of 29 PRGs between healthy and DCM samples. DCM-related PRGs were identified using univariate logistic regression, and the cut-off criterion was a *P* value < 0.05. Then, we used least absolute shrinkage and selection operator (LASSO) to improve the accuracy of the linear regression model. We utilized multivariate logistical regression to build a PRG-related DCM classifier. To evaluate the potential performance of the signature, we used receiver operating characteristic (ROC) curve analysis.

### 2.3. Correlation Analysis between PRGs and Immune Characteristics

We used the “GSVA” package to conduct single-sample gene set enrichment analysis (GSEA) to estimate the scores of infiltrating immune cells and to assess the activity of immune signalling pathways. We used the Wilcoxon test to compare enrichment fractions representing immune cell abundance and immune reactivity in healthy and DCM samples. Pearson correlation analysis was utilized to determine the relevance of PRGs with respect to immune cell components and immune response activity.

### 2.4. Identification of Pyroptosis Patterns

Based on the expression of 29 PRGs, we chose to analyse the unsupervised clustering state to identify disparate pyroptosis patterns. At the same time, we utilized a consistent clustering algorithm for the purpose of evaluating the clustering number and robustness. During the calculation, the R package “ConsensusClusterPlus” was used to perform iterative calculations. We calculated one step at a time 1000 times to ensure the robustness of the classification [20]. Moreover, to verify the expression pattern of PRGs in various pyroptosis patterns, we chose to use PCA as an analytic method after full consideration. The Kruskal test is a method that compares the degree of expression of PRGs, the score in abundance of infiltrating immunocytes, the score of immune response, and the degree of gene expression in two pyroptosis patterns, which differ widely.

### 2.5. Biological Enrichment Analysis of Distinct Pyroptosis Modification Patterns

To analyse pyroptosis-related differentially expressed genes, GO (Gene Ontology) functional enrichment and KEGG (Kyoto Encyclopedia of Genes and Genomes) pathway analyses were performed using the clusterProfiler package in R. Two sets of genes, “c5.go.v7.4.symbols” and “C2.cp.kegg.v7.4. symbols,” were used to reflect changes in biological signalling pathways. Subsequently, the expression matrix was transformed into a score matrix using the GSVA algorithm, and we used the LIMMA package to compare scores of biological signalling pathways between the two groups. The threshold of difference analysis was ADj. *P* < 0.05 and | LogFC | 1 or more.

### 2.6. Identification of Pyroptosis-Mediated Genes

To identify the genes regulating pyroptosis, we used the empirical Bayes method to analyse the samples with two different pyroptosis patterns to identify DEGs between different pyroptosis patterns. The threshold for determining important DEGs was an adjusted *P* value < 0.0001. Using weighted gene coexpression network analysis (WGCNA), we obtained the difference between gene modules and pyroptosis pattern-related genes.

### 2.7. Animal Protocol

Male C57BL/6 mice at 10 weeks of age were obtained from Hubei Provincial Centres for Disease Control and Prevention. All animal studies were conducted according to the Animal Care and Use Committee Guide of Wuhan University, which obeyed the Guide for the Care and Use of Laboratory Animals of the National Institutes of Health. To induce DCM, a cumulative dose of 12 mg/kg doxorubicin (DOX) was administered via 3 weekly IP injections (4 mg/kg on days 0, 7, and 14), and follow-up analyses were conducted 6 weeks after the first injection [17].

### 2.8. Echocardiography

All animal studies were conducted according to the Animal Care and Use Committee Guide of Wuhan University, which obeyed the Guide for the Care and Use of Laboratory Animals of the National Institutes of Health. Mice were examined using transthoracic echocardiography. Using a Vevo 2100 imaging system equipped with a 30 MHz MS400 linear array, M-mode echocardiography was performed to obtain a short-axis view of the heart at the level of the middle papillary muscle. The left ventricular systolic inner diameter (LVIDs) and left ventricular diastolic inner diameter (LVIDd) were measured in awake mice. The left ventricular ejection fraction (LVEF) was calculated as %EF = [(LVIDd − LVIDs)/LVIDd] × 100; and fractional shortening (FS) was calculated as %FS = [(LVIDd − LVIDs)/LVIDd] × 100.

### 2.9. Histological Analyses

Six weeks after the first injection, the heart tissue was collected, fixed in 4% paraformaldehyde, dehydrated, and embedded in paraffin. Four-micrometre sections were collected and stained using haematoxylin and eosin (HE) and Masson's trichrome. Images were captured using an Aperio VERSA system (Leica Biosystems, Germany) and analysed using Image-Pro Plus 6.0.

### 2.10. Real-Time PCR

Total RNA was extracted from apical tissue using TRIzol and reverse transcribed into cDNA, and real-time quantitative PCR was performed. All data are normalized to the GAPDH mRNA level as an internal reference, and the relative quantification of apical tissue mRNA expression was determined using the 2-△△CT method. The primers used are shown in Table 1.

## 3. Results

### 3.1. Expression Alterations of PRG in DCM Compared to Healthy Samples

**Figure 1:**
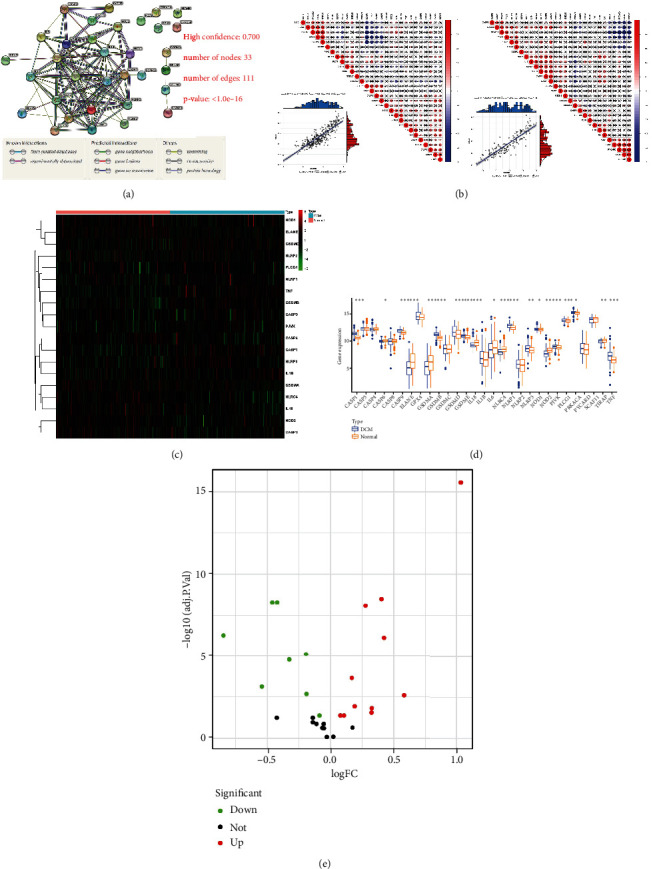
Expression landscape of PRGs in DCM. (a) The protein-protein interactions among PRGs. (b) Interrelationships of the expression of PRGs for all samples (left) and DCM samples (right). The two most interrelated PRGs shown in the two scatter plots: GSDMD and GPX4. (c) The box plot shows the expression of 19 PRGs in DCM compared to healthy samples. (d) The heat map shows the expression status of 19 PRGs in DCM compared to healthy samples. (e) The volcano plot visually shows the expression of PRGs between healthy and DCM samples.

The expression interactions of PRGs are shown in a protein–protein network (Figure 1(a)) with 111 edges and 33 nodes as determined using the STRING database. We observed that with a confidence level of 0.700, apart from PLCG1, GPX4, PRKACA, ELANE, and DFNB89, the rest of the PRGs were very closely connected. In addition, the transcriptome relationship was investigated, and we found that GSDMD and GPX4 were the most relevant PRG regulators in all samples (*r* = 0.86) and in DCM samples (*r* = 0.89), which may indicate that they work together (Figure 1(b)). At the same time, differential expression analysis identified 19 PRGs with altered expression (Figures 1(c) and 1(d)). Among them, compared to normal myocardial tissue, the fold change in TNF was the largest and most significant (Figure 1(e)).

### 3.2. PRGs Participate in the Process of DCM Generation

**Figure 2:**
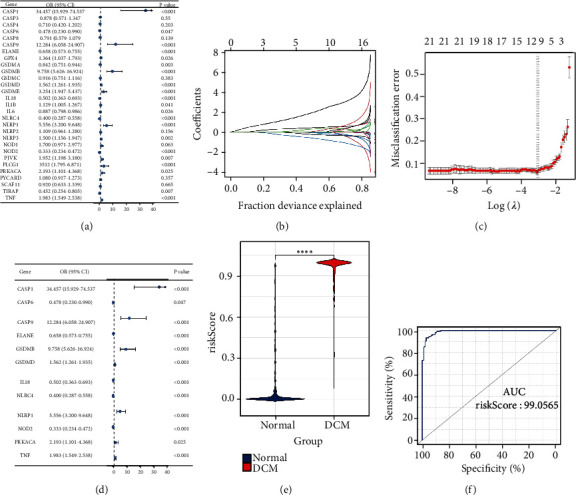
PRGs can be used to classify normal and DCM samples. (a) Univariate logistic regression was used to study the correlation between PRGs and DCM (*P* < 0.05) and uncovered 21 DCM-related PRGs. (b) LASSO coefficient distribution of 21 DCM-related RGs. (c) In LASSO regression, 10-fold cross-validation was used to fine-tune parameter selection. *λ* is the adjustment parameter, and the partial likelihood deviance is plotted according to log (*λ*). (d) Through multivariate logistic regression, a distinguishing feature with 12 PRGs was developed, and the risk scores of DCM and healthy samples were estimated. (e) The risk profile between DCM and healthy samples illustrates that the risk score of DCM was higher than that of healthy samples. (f) ROC curves were used to analyse the ability of 12 PRGs to distinguish between healthy and DCM samples, and the AUC value was used to evaluate the distinction ability.

To determine the role of PRGs in the DCM pathogenesis, we used several common bioinformatics algorithms. To identify DCM-related PRGs, we used univariate logistic regression, which showed that 21 PRGs were most closely related to DCM (Figure 2(a)). Subsequently, LASSO regression was performed on 21 DCM-related PRGs for feature selection and dimensionality reduction to exclude unimportant regulators (Figures 2(b) and 2(c)), which ultimately identified 12 hub PRGs. We used multivariate logistic regression for a classifier to distinguish between normal and DCM samples (Figure 2(d)). The classifier is composed of hub PRGs, which can well classify both normal and DCM samples based on the risk scores. The risk score of DCM was much higher than that of the normal samples (Figure 2(e)). Moreover, the ROC curve showed that the 12 PRGs could distinguish between normal and DCM samples (AUC = 0.99, Figure 2(f)). These results show that PRGs play a crucial role in the development of DCM.

### 3.3. PRGs Are Related to Immune Characteristics in DCM Tissue

**Figure 3:**
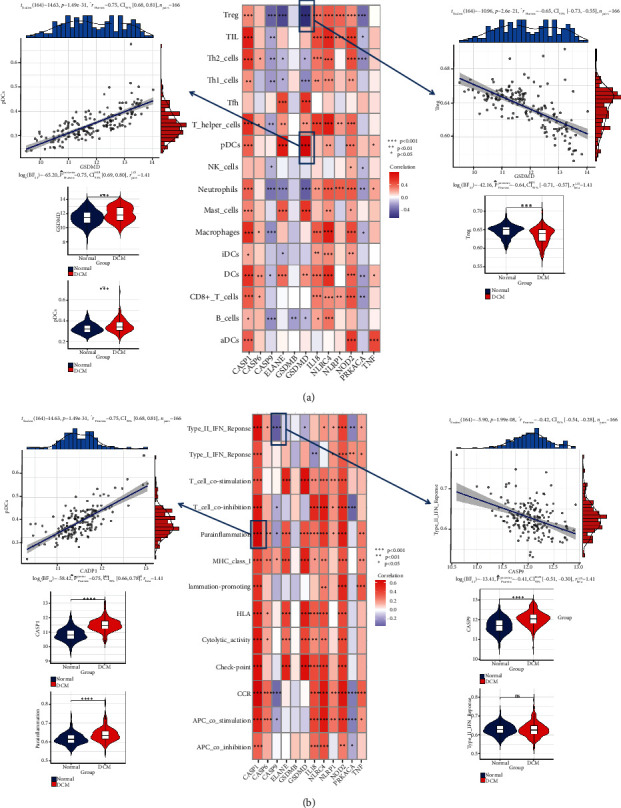
Association of PRGs with immune cells and immune response pathways. (a) The dot plot shows the correlation between each infiltrating cell and each hub PRG in the dysregulated immune microenvironment. The most positive interrelated immune cell-hub PRG pair was GSDMD-pDCs, and the violin plot on the right shows the expression or fraction status. The most negatively correlated immune cell-hub PRG pair was GSDMD-Tregs, and the violin plot on the left shows the expression or fraction status. (b) The dot plot shows the association between each gene set of the immune dysregulation response and the 12 hub PRGs. The most positively correlated pair was CASP1-parainflammation, and the violin plot in the right shows the expression or activity. The most negative interrelated pair was CASP9-type_II_IFN_response, and the violin plot on the right shows the expression or activity.

To investigate the biological behaviours between PRGs and the immune microenvironment, we analysed expression of the 12 hub PRGs, infiltrating immunocytes, and immune-related signalling pathways. Statistical analysis revealed differences in the abundance of infiltrating immunocytes between healthy and DCM samples (Figure S1A). Compared to healthy myocardial tissue, most infiltrating immune cells were altered in DCM. Correlation analysis revealed that 12 hub PRGs were closely associated with most immune cells (Figure 3(a)). For example, GSDMD had the strongest positive correlation with pDC abundance (*r* = 0.75) and the strongest negative correlation with Treg abundance (*r* = −0.65), which was related to the expression status of GSDMD, pDCs, and Tregs. The box plot shows differences in the activity of each immune response pathway between healthy and DCM samples (Figure S1B). In addition, we found that CASP1 was positively correlated with parainflammation (*r* = 0.73) and that CASP9 was negatively correlated with the type II IFN response (*r* = −0.42) (Figure 3(b)). This indicates that CASP1 and CASP9 play important roles in parainflammation or type II IFN response in DCM.

### 3.4. PRG-Mediated Pyroptosis Patterns in DCM

**Figure 4:**
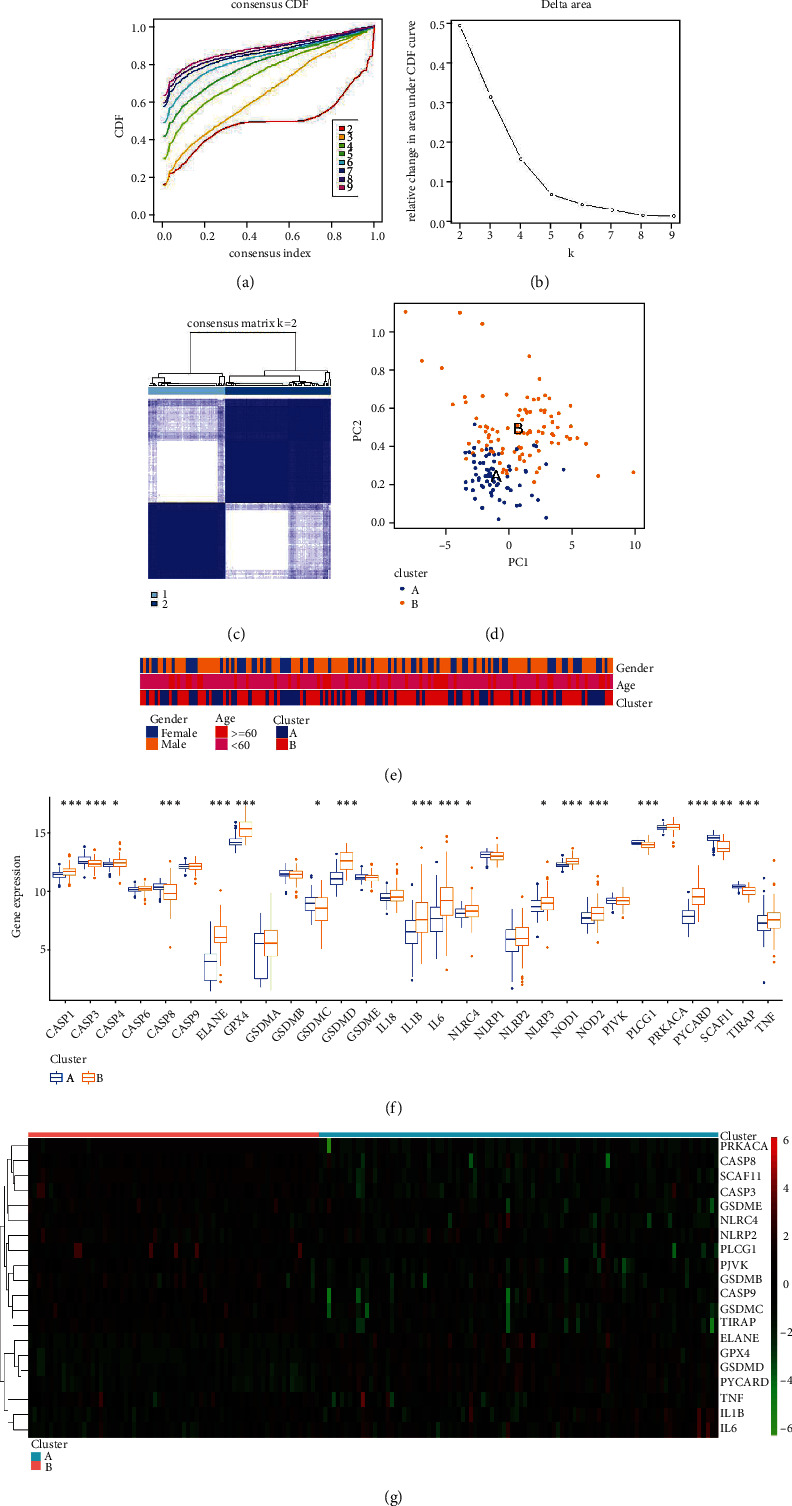
Identification two different subtypes of pyroptosis patterns in DCM. (a) A consensus clustering cumulative distribution function (CDF) with *k* = 2 − 7 is shown. (b) The relative change in the area under the CDF curve for *k* = 2 − 7 is shown. (c) Heat map of the matrix of cooccurrence proportions for DCM samples. (d) Principal component analysis (PCA) of the transcriptome profiles of the two pyroptosis patterns, indicating that there were significant differences in the transcriptome between the two pyroptosis patterns. (e) Comparison of age and sex between the two pyroptosis patterns. The correlation of different clinical features between the 2 patterns is shown by the heat map. (f) Box plot of the expression status of PRGs in two pyroptosis patterns. (g) Heat map of the expression status of PRGs in the two pyroptosis patterns.

Based on the expression of 29 PRGs, we performed unsupervised consensus clustering analysis for DCM samples to investigate pyroptosis patterns in DCM (Figures 4(a)–4(c)). Two distinct DCM subtypes were identified. PCA showed that expression of PRGs was qualitatively different between the subtypes, including 70 samples in subtype A and 96 samples in subtype B (Figure 4(d)). In addition, correlation analysis indicated no significant difference in clinical characteristics between the different patterns (Figure 4(e)). Except for CASP6, CASP9, GSDMA, GSDMB, GSDME, IL18, NLRP1, NLRP2, PJVK, PRKACA, and TNF, expression of the remaining 19 PRGs in different pyroptosis patterns exhibited obvious differences (Figures 4(f) and 4(g)). Multiple pyroptosis patterns were verified in DCM.

### 3.5. Characteristics of the Immune Microenvironment in Different Pyroptosis Patterns

**Figure 5:**
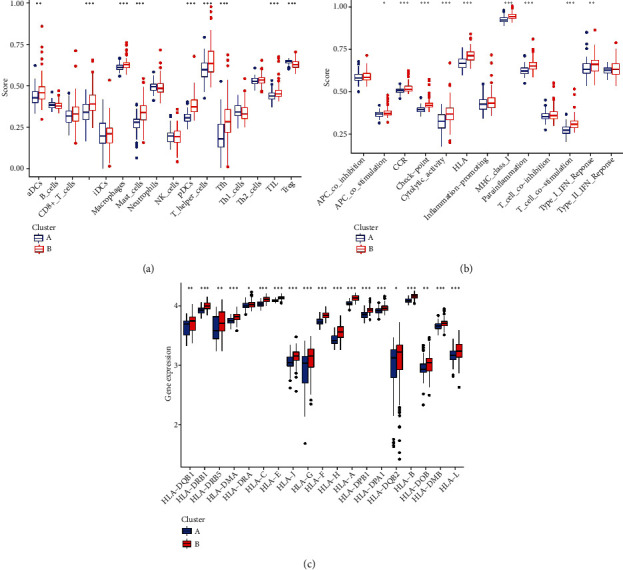
Differences in immune microenvironmental features in different pyroptosis patterns. (a) The abundance difference of infiltrating immunocytes in each immune microenvironment in pyroptosis patterns. (b) Activity differences in each immune response gene set in pyroptosis patterns. (c) Expression differences in each HLA gene in the pyroptosis patterns.

To identify differences in immune microenvironmental characteristics between these different pyroptosis patterns, we evaluated immune cells, immune response gene sets, and human leukocyte antigen (HLA) gene expression. Many immune cells were different between the two patterns (Figure 5(a)). Compared to pattern B, pattern A had relatively few infiltrated immunocytes. Pattern B displayed higher levels of aDCs, DCs, macrophages, mast cells, NK cells, T helper cells, Tfhs, and TILs. Of note, only Treg cells were more enriched in pattern A. In addition, in terms of immune response, the immune response of pattern B was more active. For instance, the immune response of MHC class I and HLA was very active in pattern B (Figure 5(b)). At the same time, we observed a similar trend in the gene expression of HLA (Figure 5(c)). These results indicated that pyroptosis pattern B mediates a more active immune response, while the pyroptosis pattern A-mediated immune response is relatively mild. These results once again strongly demonstrated that pyroptosis has an important regulatory effect on the formation of different immune microenvironments of DCM

.

### 3.6. Biological Characteristics of Pyroptosis Patterns

**Figure 6:**
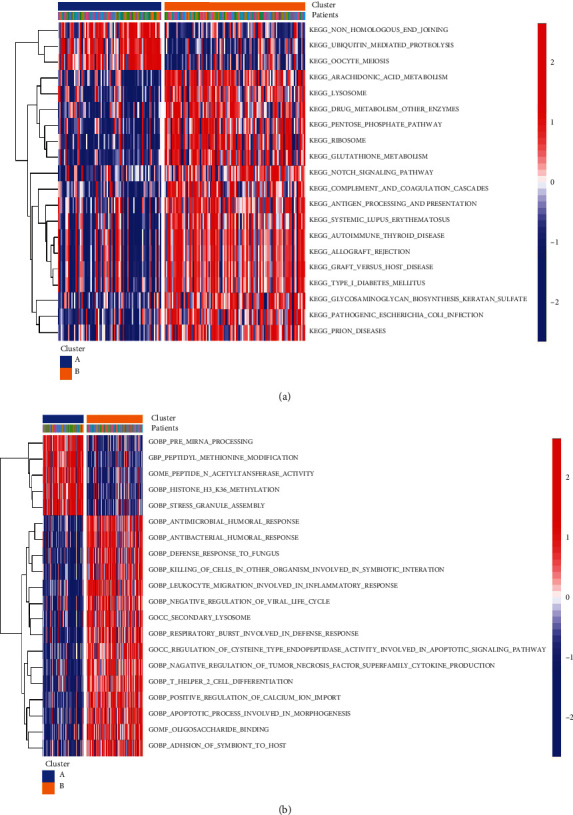
Diversity of potential biological function characteristics between the two pyroptosis patterns. (a) Differences in KEGG pathway enrichment scores between the two pyroptosis patterns. (b) Differences in GO pathway enrichment scores between the two pyroptosis patterns.

**Figure 7:**
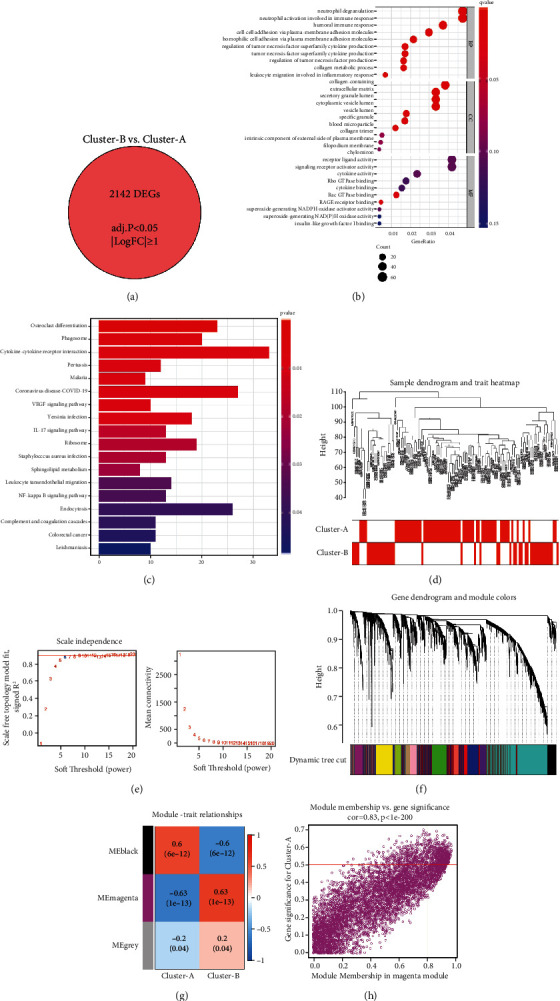
Identification and functional analysis of genes involved in the pyroptosis phenotype in DCM. (a) 2142 genes were related to the pyroptosis phenotype. (b) GO-BP functional enrichment analysis showed the biological features of genes involved in the pyroptosis phenotype. (c) GO enrichment analysis of genes related to the pyroptosis phenotype revealed the correlation between PRGs and immune mechanisms. (d) Sample clustering was performed on the strength of the expression information of all samples. All differentially expressed genes with *P* < 0.05 were used for WGCNA. (e) Analysis of the scale-free ft index and analysis of the mean connectivity for various soft-thresholding powers. (f) Gene tree diagram. The coloured line below the tree diagram shows the modules determined using dynamic tree cutting. (g) Heat map of the relevance between the characteristic genes of the module and the pyrolysis pattern. (h) Gene significance (GS) scatter plot of pyrolysis pattern A and module member (MM) in the magenta module.

To investigate the biological response of the pyroptosis patterns, GO analysis and KEGG analysis were performed. We applied GSVA enrichment analysis to estimate the activity of the biological pathways that were assessed. In the KEGG pathway analysis, compared to pattern A, pattern B had more abundant pathways, such as the NOTCH signalling pathway and pentose phosphate pathway (Figure 6(a), Supplementary File 1). In GO pathway analysis, compared to pattern A, pattern B also had more abundant pathways, such as antimicrobial humoral response and other biological processes (Figure 6(b), Supplementary File 2). To investigate the mechanism of genes related to PRG-mediated regulation, we identified DEGs related to the pyroptosis phenotype. A total of 2142 common genes were considered to be related to the pyroptosis phenotype (Figure 7(a)), and GO enrichment analysis showed that they were primarily involved in immune processes such as neutrophil activation in the immune response (Figure 7(b)). In addition, in the KEGG analysis, the selected biological process of DEG enrichment was significantly related to biological processes such as cytokine–cytokine receptor interaction (Figure 7(c)). In addition, we used WGCNA to identify gene–gene modules related to different modifications (Figures 7(d)–7(f)). We identified three gene modules where different pyroptosis patterns matched their related genes (Figure 7(g)); for example, pyroptosis pattern A was closely related to genes in the magenta module (Figure 7(h)). The above results could clarify that pyroptosis patterns mediate the related gene expression regulation network.

### 3.7. Validation of the Expression Levels of Five Core PRGs in DCM

**Figure 8:**
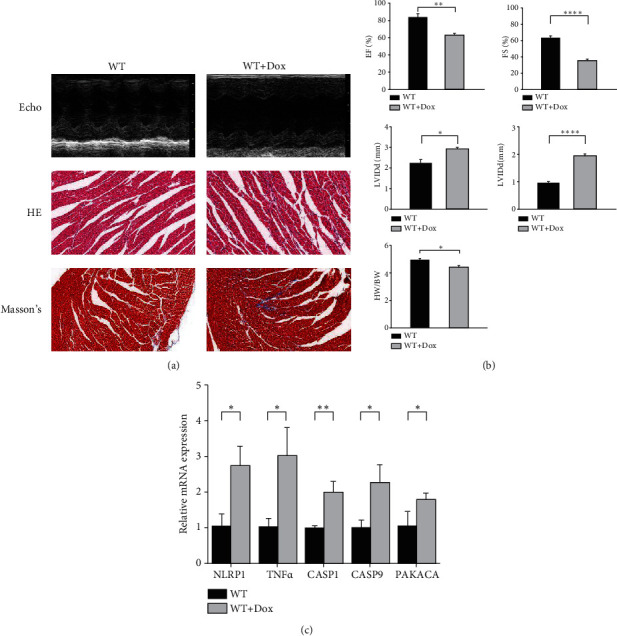
Validation of the expression levels of NLRP1, TNF*α*, CASP1, CASP9, and PRKACA in healthy and Dox-induced DCM mouse myocardial tissues. (a) Representative echocardiographic (echo), HE, and Masson's trichrome staining images. (b) Cardiac function index (*n* = 6). (c) Expression levels of NLRP1, TNF*α*,CASP1, CASP9, and PRKACA were quantified using qRT–PCR analysis in myocardial tissue. ^∗^*P* < 0.05, ^∗∗^*P* < 0.01, ^∗∗∗^*P* < 0.001, and ^∗∗∗∗^*P* < 0.0001.

According to the bioinformatics results, we further validated expression of the five core PRGs (NLRP1, TNF*α*, CASP1, CASP9, and PRKACA) in healthy mouse myocardial tissue and Dox-induced DCM myocardial tissue (Figures 8(a) and 8(b)). The RT–PCR results showed that the mRNA expression of NLRP1, TNF*α*, CASP1, CASP9, and PRKACA in DCM myocardial tissues was significantly higher than that in normal myocardial tissues (*P* < 0.05), consistent with the results of bioinformatics analysis (Figure 8(c).

## 4. Discussion

Dilated cardiomyopathy (DCM) is considered the final common reaction of the myocardium due to a combination of genetic and environmental factors. Pyroptosis was initially found to be a key mechanism for fighting against infection [22–24]. Recently, extensive findings have indicated that pyroptosis is involved in various cardiovascular diseases (CVDs) [15, 25, 26]. However, it is unknown whether the pyroptosis signalling pathway is involved in the immune microenvironment regulation of dilated cardiomyopathy. In this study, pyroptosis patterns were found in the immune response of DCM. To understand how PRG mediates the immune response and the alteration of immune cells in DCM, we used multiple bioinformatics analyses to obtain these results. First, our results identified 19 PRGs with altered expression between healthy and DCM samples, indicating that PRGs do indeed participate in the development of DCM. Based on hub DCM-related PRGs, we developed a classifier to differentiate between normal samples from DCM samples. The classifier performed well in distinguishing between healthy and DCM samples, revealing that PRGs do indeed play a critical role in the development of DCM. The mRNA expression levels of core PRGs, which are upregulated in DCM tissues, were indeed higher than that of healthy tissues. This was verified using qRT–PCR analysis in mouse myocardial tissue. In addition, our findings revealed that differences in the abundance of immune cells do indeed exist in the immune microenvironment between healthy and DCM samples. CASP1 was positively related to parainflammation, and CASP9 was negatively related to the type II IFN response. This indicates that CASP1 and CASP9 play important roles in parainflammation or the type II IFN response in DCM. In addition, we found that gasdermin D (GSDMD) was strongly positively correlated with pDC abundance. GSDMD was recently identified as the factor responsible for the inflammatory form of lytic pyroptotic cell death, a critical antibacterial innate immune defence mechanism [27–29]. GSDMD is pleiotropic, exerting both pro- and anti-inflammatory effects, which make it a potential target for antibacterial and anti-inflammatory therapies [27, 30]. These findings may indicate the existence of a PRG immune regulatory mechanism in DCM. To investigate pyroptosis patterns in DCM, unsupervised consensus clustering analysis was conducted for DCM samples based on the expression of 29 PRGs. Two distinct DCM subtypes were identified. In addition, correlation analysis showed that there was no significant difference in clinical characteristics between different pyroptosis patterns. Expression of most PRGs in different pyroptosis patterns displayed obvious differences. It was verified that there were multiple pyroptosis patterns in DCM. Compared to pattern B, pattern A exhibited relatively few infiltrated immunocytes. Pattern B presented higher levels of aDCs, DCs, macrophages, mast cells, NK cells, T helper cells, Tfhs, and TILs, while Tregs were more highly enriched in pattern A. In addition, the immune response of pattern B was more active. For instance, the immune response of MHC class I and HLA is active in pattern B. We identified a similar trend in HLA gene expression. These results indicated that pyroptosis pattern B is characterized by a more active immune response, while pyroptosis pattern A features are relatively mild immune response. These results once again strongly demonstrate that cell pyroptosis exerts an important regulatory effect on the formation of different immune microenvironments of DCM. Moreover, compared to pattern A, pattern B contained more abundant pathways, such as the NOTCH signalling pathway, pentose phosphate pathway, antimicrobial humoral response, and other biological processes. These results regarding biological response clarify the gene expression regulation network mediated by pyroptosis patterns. This study provides some important findings for the exploration of cell pyroptosis in DCM to facilitate characterization of pyroptosis mechanisms and immune characteristics in DCM by other investigators.

Abundant results were generated that will promote our understanding of immune mechanisms with respect to pyroptosis in DCM. In addition, we identified two different patterns of pyroptosis, and they can help us deepen our comprehension of pyroptosis in DCM to understand how it mediates the immune response. According to our inferences, the correlation between cell pyroptosis and the immune microenvironment is strong and significant.

However, our study still has some limitations that need to be considered. First, the number of samples included in this study was limited, so further experiments are needed to confirm the results of this study. Second, all data in this study are based on the expression values of mRNA and do not directly reflect protein expression levels, which may result in poor performance in the evaluation of immune signal pathways based on protein expression. For instance, inconsistency between the molecular pathways is directly related to cellular activities. Although such limitations should not be ignored, our results suggest that pyroptosis has a significant influence on the immune microenvironment in DCM and deepens our understanding of the potential pathogenesis of DCM.

Our findings systematically revealed the potential association between cell pyroptosis and the immune microenvironment in DCM. Through our research, it was confirmed that pyroptosis is closely related to DCM and that pyroptosis has a regulatory effect on the immune microenvironment in DCM. The findings could provide ideas for other researchers in the field to further investigate the mechanisms of pyroptosis in DCM. We screened out genes that are closely related to pyroptosis in DCM. By regulating these genes to inhibit pyroptosis, these genes are likely to become potential targets for new therapeutic interventions and play a role in relieving or treating DCM. In addition, the mechanism of DCM is very complicated, and it is not yet fully understood. Through studying the relationship between pyroptosis and the immune environment, we can gain a deeper understanding of the immune mechanism of DCM. Through studying the molecular pathways of pyroptosis in DCM, these results may offer clues for new therapeutic strategies for DCM. We believe that the investigation of pyroptosis in the immune microenvironment in DCM may be meaningful in the future.

## 5. Conclusions

In summary, pyroptosis plays a critical role in the immune regulation of dilated cardiomyopathy. The study demonstrated that pyroptosis has an important regulatory effect on the formation of different immune microenvironments by impacting infiltrating immunocytes. This study offers novel ideas for understanding the pathogenesis of dilated cardiomyopathy, which will be very significant in the future.

## Figures and Tables

**Table 1:** Primers used for RT-PCRs.

Target genes	Forward primers (5′-3′)	Reverse primers (5′-3′)
NLRP1	AGCAAGGGTGGAACAGCATT	ATAGCGGGAACCAAGATAAAGAG
TNF*α*	CTCTTCTGTCTACTGAACTTCGGG	GGTGGTTTGTGAGTGTGAGGGT
CASP1	GGCTGACAAGATCCTGAGGG	TAGGTCCCGTGCCTTGTCC
CASP9	GAGGTGAAGAACGACCTGACTG	CTCAATGGACACGGAGCATC
PRKACA	ATCGTCCTGACCTTTGAGTATCTG	ACAGCCTTGTTGTAGCCTTTGC
GAPDH	CCTCGTCCCGTAGACAAAATG	TGAGGTCAATGAAGGGGTCGT

## Data Availability

https://www.ncbi.nlm.nih.gov/geo/query/acc.cgi?acc=GSE141910.

## Supplementary Materials

Supplementary Materials Figure S1: differential expression of infiltrating immune cells and immune response pathways.
