# Supplementary figures and images for: Pyroptosis Patterns Are Involved in Immune Microenvironment Regulation of Dilated Cardiomyopathy
Source: Dis Markers. 2022 Mar 10;2022:4627845. doi: 10.1155/2022/4627845 (PMC8930255; doi:10.1155/2022/4627845)

**A**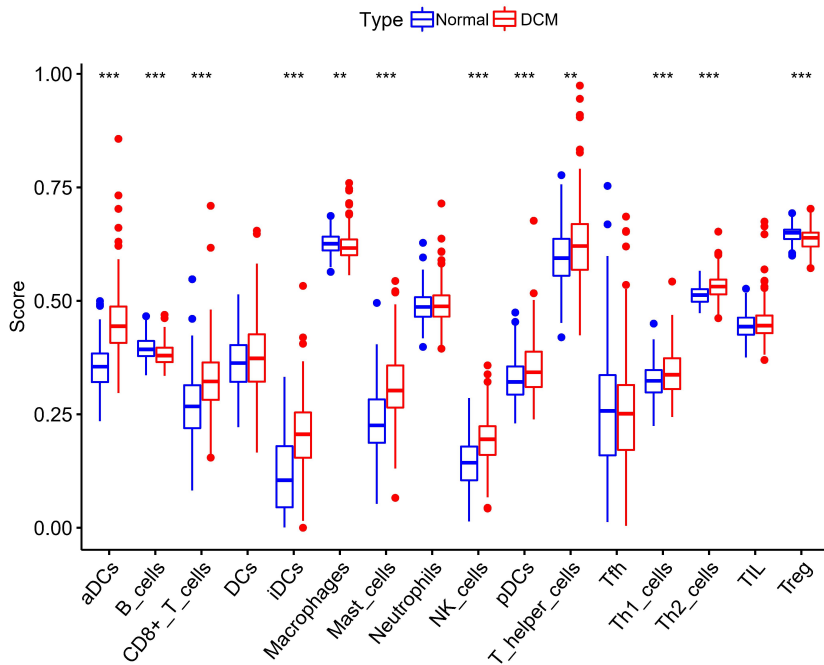**B**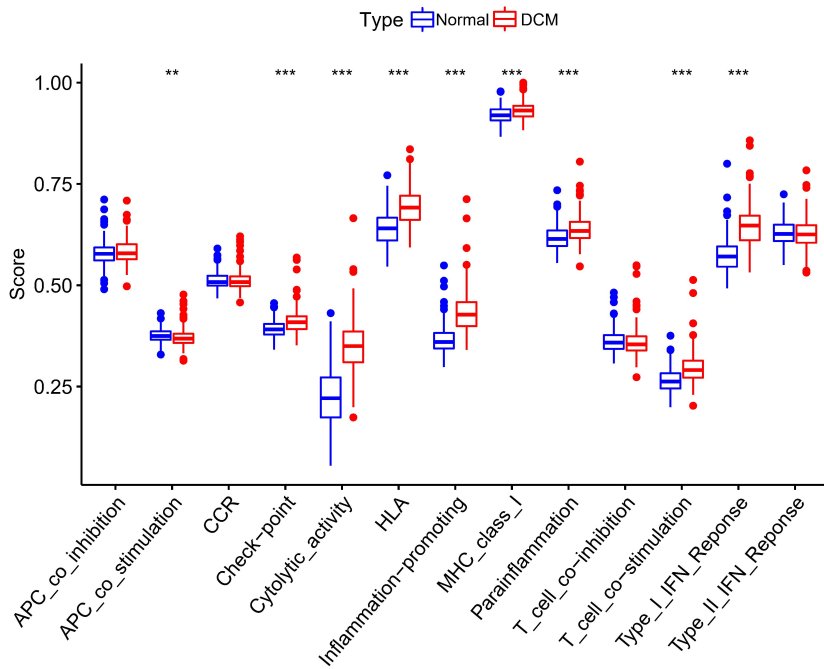

Supplement: Supplementary Materials — Figure S1: differential expression of infiltrating immune cells and immune response pathways. [file 4627845.f1.pdf]
